# The therapeutic efficacy of *Aloe vera* gel ointment on staphylococcal pyoderma in dogs

**DOI:** 10.14202/vetworld.2020.2371-2380

**Published:** 2020-11-09

**Authors:** Ahmed Kamr, Ali Arbaga, Amanallah El-Bahrawy, Ahmed Elsify, Hadeer Khaled, Hany Hassan

**Affiliations:** 1 Department of Animal Medicine and Infectious Diseases, Faculty of Veterinary Medicine, University of Sadat City, Sadat City, Egypt; 2 Department of Veterinary Pathology, Faculty of Veterinary Medicine, University of Sadat City, Sadat City, Egypt

**Keywords:** catalase, dogs, interleukins, malondialdehyde, pyoderma, serum amyloid A

## Abstract

**Background and Aim:**

Staphylococcus pyoderma is a common problem in dogs that need a novel treatment rather than antibiotic therapy. The aim of this study was to investigate the therapeutic efficacy, anti-inflammatory, and antioxidative properties of *Aloe vera* (*Aloe barbadensis*) gel ointment on dogs’ Staphylococcus pyoderma compared to gentamicin ointment.

**Materials and Methods:**

The inhibition zone of *A. vera* extract 20% and 40% and gentamicin 1% against *Staphylococcus aureus* was determined on well diffusion agar. Twenty Baladi local breed dogs were used as control negative group before intradermal inoculation with 10^5^ CFU *S. aureus*. The animals were classified into four equal groups, control positive group without treatment (n=5), treated groups by 20% *A. vera* gel ointment (n=5), 40% *A. vera* gel ointment (n=5), and gentamicin ointment 1% (n=5). Topical application of *A. vera* and gentamicin ointments was carried out twice daily for 2 weeks until complete healing of dogs’ pyoderma. Clinical evaluation was recorded. Inflammatory, oxidant, and antioxidant parameters were measured in serum.

**Results:**

The inhibition zone of *A. vera* extracts 20% and 40% was 19 mm and 23 mm, respectively, while gentamicin 1% was 18 mm. The half maximal inhibitory concentration (of *A. vera* 20% and 40% were 13.70 with R^2^=0.98. Dogs’ pyoderma treated with *A. vera* gel ointment 20% and 40% were more likely to have low haptoglobin and tumor necrosis factor-α concentrations than gentamicin 1% ([odds ratio [OR]=4.6; 95% confidence interval [CI]=1.31-17.40; p<0.05]; [OR=5.2; 95% CI=1.04-22.30; p<0.05]), respectively.

**Conclusion:**

It seems evident that *A. vera* has therapeutic effect, antibacterial, and anti-inflammatory effects against dogs’ staphylococcal pyoderma than gentamicin that would support its further use rather than antibiotics in one health arena.

## Introduction

*Aloe vera* (*Aloe barbadensis*) is a medicinal plant that has many functions include wound healing, immunomodulation, and hepatoprotection in addition to its antiviral, skin protective, antioxidant, antidiabetic, anti-inflammatory, antimicrobial, and anticancer properties [1]. *A. vera* is characterized by the presence of many active compounds, inclusive of amino acids, minerals, anthraquinones, chromones, nutrients, lipids, anthrones, carbohydrates, and flavonoids [2]. Canine superficial pyoderma is described as superficial bacterial contamination of the dermis and hair follicles that caused by *Staphylococcus* spp. [3]. Pyoderma can be caused by infectious, neoplastic, and/or inflammatory etiologies with consequences of the accumulation of neutrophilic exudates and associated with increased free radical production, oxidative stress [4,5]. Of interest, *Staphylococcus aureus*, which is considered methicillin-resistant, was isolated from dogs with recurrent pyoderma [6].

Acute-phase response is a distinguished systemic reaction of the body within 24-48 h after local or systemic diseases due to infection, tissues damage, surgery, trauma, and immunological impairment [7-9]. Serum amyloid A (SAA), haptoglobin (HP), and ceruloplasmin (CP) were produced by liver and considered prognostic biomarkers for acute inflammation in dogs as a result of tissue infection or inflammation [10,11]. Cytokines are small glycoproteins (~5–20 kDa) that have autocrine, paracrine, and endocrine signaling. Cytokines include chemokines, interferons, interleukins (IL), lymphokines, and tumor necrosis factors (TNF) are produced by many types of cells that include macrophages and lymphocytes that are essential in host immune responses to infection, inflammation, trauma, sepsis, cancer, and reproduction [12]. However, the inflammatory and immunomodulatory roles of *A. vera* in dogs’ pyoderma need further investigation.

This study investigated the beneficial healing effect, antibacterial, anti-inflammatory, and antioxidative roles of *A. vera* in dogs’ staphylococcal pyoderma. We hypothesized that acute-phase proteins, cytokines, and oxidants will be elevated, while antioxidant biomarkers will be reduced in dog’s staphylococcal pyoderma; furthermore, *A. vera* gel ointment will modulate the clinical image inflammatory response in a better manner than gentamicin against staphylococcal pyoderma in dogs.

## Materials and Methods

### Ethical approval

All dogs were used and treated according to Animal Ethics Committee at College of Veterinary Medicine, University of Sadat City (Approval code VUSC-007-1-19).

### Study period and location

This study was conducted between September 2018 and May 2019. The experiment was conducted at Animal Medicine and Infectious Diseases Department, teaching hospital and laboratory, University of Sadat City.

### Animals’ criteria

A total of 20 Baladi local male adult dogs 2-3 years old were housed in individual cages for acclimatization for 3 weeks, with free access to food and water. According to the production company Pharma Swede, Egypt, dogs were dewormed by subcutaneous injection of ivermectin (Paramectin^®^) at a dosage of 10 mg/50 kg as a routine protocol against external parasites that would interfere with experimental induction of canine pyoderma.

### Experimental design

Twenty male adult dogs of Baladi local breed were clinically healthy based on their physical examination. The animals were considered as a control negative group before induction of pyoderma (n=20). The dogs were inoculated intradermally in chest region with 1 mL broth containing 10^5^ CFU staphylococcus [6,13]. The injected dogs were further classified into further subgroups after appearance of pyoderma lesions into control positive group without treatment (n=5), treated groups by 20% *A. vera* gel ointment (n=5), 40% *A. vera* gel ointment (n=5), and gentamicin ointment 0.1% (n=5). Topical application of *A. vera* and gentamicin ointments (Garamycin^®^ 0.1%; Schering-Plough Company, USA) was carried twice daily for 2 weeks until complete healing of dogs’ pyoderma.

### *In vitro* evaluation of antibacterial effect of *A. vera* extract

Antibacterial activity of *A. vera* extract was evaluated using agar diffusion test in which 15-20 mL of nutrient agar (Oxoid^®^) was poured on glass Petri plates of same size and allowed to solidify. Agar surface of each plate was streaked by a sterile cotton swab with *S. aureus* equivalent to 0.5 McFarland standards. Agar plate was punched with a sterile cork borer of 4 mm size; then, *A. vera* fresh extract 20% and 40% concentration, gentamycin 0.1%, pure *A. vera*, and pure ethanol were filled into its wells. The plate was allowed to stand by for 30 min. The plate was incubated at 37°C for 48 h and examined. The diameter of inhibition zones was measured [14].

### *A. vera* gel ointment 20% and 40% preparation

Preparation of *A. vera* gel ointments 20% and 40% formula was carried out by adding 20 g and 40 g of *A. vera* gel, respectively, 3 g of wax and 5 mL of paraffin oil with 72 g and 52 g of Vaseline™, respectively. All components were mixed well into mortar until obtaining homogenous ointments. Gentamycin ointment was purchased as a commercial product from the pharmacy (Garamycin^®^ 0.1%; Schering-Plough Company, USA). *A. vera* concentrations of 5%, 10%, and 15% did not give the optimal results against dogs’ pyoderma, so we used 20% and 40% *A. vera* concentrations in this study.

### Sampling

Blood samples were collected from admitted dogs in EDTA and serum clot tubes for hematobiochemical analysis before induction and on 3^rd^, 7^th^, 10^th^, and 14^th^ day after induction and treatment of Staphylococcus pyoderma. Samples were centrifuged at 2000× *g* for 10 min at 4°C. Serum and plasma were aliquoted into smaller volumes and stored at −80°C until analyzed.

### Clinical examination and complete blood count

Physical and clinical examinations were determined for all dogs according to methods specified by Englar [15]. The hemogram and leukogram examinations were measured and calculated for each dog according to methods described by Turgeon [16]. Dog’s pyoderma lesions diameter (Ø) was measured and expressed as mm.

### Oxidant, antioxidant, and inflammatory biomarkers measurements

Serum malondialdehyde (MDA), total antioxidant capacity (TAC), and catalase concentrations were measured by commercial kits (Bio Diagnostics, Ltd., Egypt) according to methods described by Ohkawa *et al*. [17], Koracevic *et al*. [18], Aebi [19], respectively. Serum CP, SAA, HP, and ILs; IL-1, IL-6, and IL-10 and TNF-α were determined by commercial ELISA kits (Shanghai Coon Koon Biotech., Ltd.; China) with inter- and intra-coefficient of variations <10 and good linearity (R^2^=0.95). All procedures were performed as described in the instructions of the manufactures.

### Statistical analysis

Data were assessed for normality and were normally distributed based on Shapiro–Wilk test. Data were expressed as mean with standard error. Comparisons between the groups were performed by one-way ANOVA using SPSS Statistics 24.0 version [20]. Receiver operating characteristic curve and the half maximal inhibitory concentration (IC_50_) were calculated using GraphPad Prism 8 (GraphPad Software, Inc., La Jolla, CA, USA). Univariate logistic regression includes odds ratio (OR) and 95% confidence interval (CI) was calculated. Significance was set at p<0.05.

## Results

### *In vitro* evaluation of antibacterial effect of *A. vera* extracts and drug efficacy on dogs’ staphylococcal pyoderma

**Figure-1:**
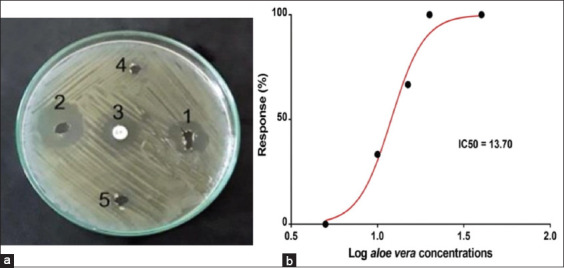
*In vitro* evaluation of antibacterial effect of *Aloe vera* extracts and drug efficacy on dogs’ staphylococcal pyoderma. (a) *In vitro* evaluation of antibacterial activity of different concentrations *A. vera* extracts 20% (1) and 40% (2), gentamicin (3), raw *A. vera* gel (4), and pure ethanol (5); (b) *A. vera* concentrations 20% and 40% had half maximal inhibitory concentration (IC_50_) than *A. vera* 5%, 10%, and 15% concentrations (IC_50_=13.70; 95% confidence interval=12.54-14.88 with R^2^=0.98).

*A. vera* 20% and 40% extracts and gentamycin 0.1% inhibition zone diameter were 19 mm, 23 mm, and 18 mm, respectively, compared by raw *A. vera* gel and pure ethanol, as shown in Figure-1a. *A. vera* concentrations 20% and 40% had IC_50_=13.70 with 95% CI=12.54-14.88 with R^2^=0.98; p<0.05; (Figure-1b).

### Clinical findings between the studied groups of dogs

**Figure-2:**
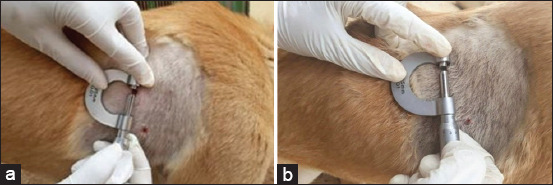
Measurement of pyoderma lesion diameter (ø) in a dog topically treated with Aloe vera gel ointment 40% (a) lesion ø 6 mm on the 3^rd^ day post-treatment (DPT); (b) lesion ø 3 mm at the 7^th^ DPT.

**Figure-3:**
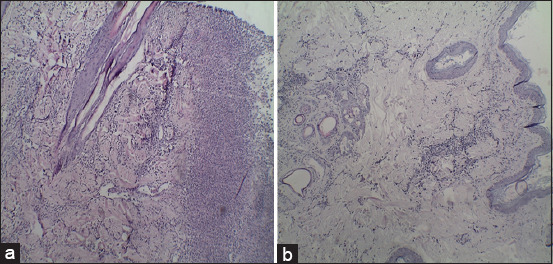
Histopathological findings. (a) Control positive group was showing sloughing of epidermis, severe inflammatory cells infiltration in the dermis, necrosis, and collagen degeneration; (b) *Aloe vera* treated group with concentration of 40% was showing normal epidermis and few inflammatory cells infiltration in the dermis on the 14^th^ day post-treatment.

There was a significant increase in diameter of pyoderma lesions in infected dogs on 3^rd^-10^th^ day without treatment compared to control negative group (p<0.05; Table-1). In dogs treated with *A. vera* gel ointment 20% and 40% and gentamicin ointments 0.1%, the diameter of the pyoderma lesions was significantly reduced on the 3^rd^ and 7^th^ day post-treatment (DPT) compared to control positive group dogs (p<0.05; Figure-2a and b and Table-1), while there is no statistical variation between dogs treated with *A. vera* gel ointment 20% and 40% and gentamicin ointments 0.1% and control ones (p>0.05; Table-1). Control positive group with Staphylococcus pyoderma showed sloughing of epidermis, severe inflammatory cells infiltration in the dermis, necrosis, and collagen degeneration (Figure-3a). *A. vera* gel ointment treated group with concentration of 40% showed normal epidermis and few inflammatory cells infiltration in the dermis on the 14^th^ day of treatment (Figure-3b). The mean values of body temperature, respiratory, and pulse rates were elevated in infected dogs on the 3^rd^-10^th^ day without treatment compared to healthy control negative dogs (p<0.05; Table-2), but not with the 14^th^ day without treatment (p>0.05). There was significant increase for the values of body temperature, respiratory, and pulse rates in dogs treated with *A. vera* gel ointment 20% and 40% and gentamicin ointment 0.1% at different times of treatment on the 3^rd^ DPT (p>0.05; Table-2), then returned to their normal values from the 7^th^ to 14^th^ DPT when compared to control healthy ones (p>0.05; Table-2).

**Table-1:** Caliber measurement of dog’s pyoderma lesions diameter (Ø) (mm). Values expressed as mean with SE.

Variables	Ø of pyoderma lesion (mm)
Control negative dogs (before induction) (n=20)	No lesions
Infected dogs without treatment Group 1 (n=5)	
3 DWT	8.12±0.17^b^
7 DWT	6.1±0.2^c^
10 DWT	4.3±0.28^d^
14 DWT	2.2±0.18^e^
Dogs treated with *Aloe vera* gel ointment 20% Group 2 (n=5)	
3 DPT	6±0.2^c^
7 DPT	3.8±0.2^d^
10 DPT	Cured
14 DPT	Epidermal collarette (lesion with a circular scaly edge)
Dogs treated with *Aloe vera* gel ointment 40% Group 3 (n=5)	
3 DPT	6±0.3^c^
7 DPT	3.6±0.4^d^
10 DPT	Epidermal collarette (lesion with a circular scaly edge)
14 DPT	Complete healing
Dogs treated by gentamicin sulfate 0.1% ointment Group 4 (n=5)	
3 DPT	6.48±0.4^c^
7 DPT	3.85±0.11^d^
10 DPT	Cured
14 DPT	Epidermal collarette (lesion with a circular scaly edge)

SE=Standard error, DWT=Day without treatment, DPT=Day post-treatment, n=Number, mm=Millimeter. Means with different letter superscripts in the same column are significantly different at p<0.05

**Table-2:** Clinical examination of dogs. Values expressed as mean with SE.

Variables	Body temperature (°C)	Respiratory rate (cycle/min)	Pulse rate (beat/min)
Control negative dogs (before induction) (n=20)	37.96±0.14^a^	24.6±1.3^a^	80±1.7^a^
Infected dogs without treatment Group 1 (n=5)			
3 DWT	38.9±0.1^b^	32.5±1.04^b^	91.25±1.49^b^
7 DWT	38.4±0.08^c^	29±1.29^c^	85±0.4^c^
10 DWT	38.35±0.2^c^	27.75±1.03^c^	84.25±0.85^c^
14 DWT	38.2±0.85^a^	26±0.4^a^	82.4±1.6^a^
Dogs treated with *Aloe vera* gel ointment 20% Group 2 (n=5)			
3 DPT	38.7±0.24^b^	28.6±1.8^c^	85.3±2.06^c^
7 DPT	38.2±0.1^a^	25.4±0.8^a^	83.4±0.8^a^
10 DPT	38.3±0.4^a^	26±1.4^a^	82±1.2^a^
14 DPT	38.2±0.08^a^	25.2±1.06^a^	80.8±1.4^a^
Dogs treated with *Aloe vera* gel ointment 40% Group 3 (n=5)			
3 DPT	38.6±0.2^b^	29.5±0.6^c^	86.4±1.8^c^
7 DPT	38.2±0.14^a^	24.8±1.54^a^	82±1.34^a^
10 DPT	37.8±0.3^a^	25±0.8^a^	80.6±0.88^a^
14 DPT	38±0.2^a^	23.3±1.6^a^	81.4±2.08^a^
Dogs treated by gentamicin sulfate 0.1% ointment Group 4 (n=5)			
3 DPT	38.6±0.09^b^	30.2±0.8^c^	88.4±0.85^c^
7 DPT	38±0.4^a^	26.3 ±1.2^a^	80.4±2.4^a^
10 DPT	38.2±0.1^a^	24.2±0.8^a^	82±0.88^a^
14 DPT	37.8±0.2^a^	25.5±1.4^a^	82.5±1.4^a^

SE=Standard error, DWT=Day without treatment, DPT=Day post-treatment, n=Number. Means with different letter superscripts in the same column are significantly different at p<0.05

### Hematological findings between the studied groups of dogs

The mean values of the red blood cells (RBCs) and hemoglobin (Hb) were significantly decreased in infected control positive dogs on the 3^rd^-14^th^ day without treatment compared to control negative ones (p<0.05; Table-3). Packed cell volume (PCV) values were significantly decreased in infected control positive dogs on the 3^rd^-10^th^ day without treatment compared to control negative ones (p<0.05; Table-3), but not on the 14^th^ day without treatment (p>0.05). In *A. vera* ointment gel 20% treated group, RBCs and Hb values were decreased on the 3^rd^ DPT (p<0.05), then returned to their baseline on the 7^th^-14^th^ DPT when compared to control ones (p>0.05). No significance was recorded in PCV values between groups treated with *A. vera* gel ointment 20% and healthy ones (p>0.05). No significant change for RBCs values in *A. vera* 40% treated group at different time points of treatment when compared with healthy ones before induction (p>0.05). In *A. vera* 40% treated group, Hb values were decreased on the 3^rd^ DPT, while PCV values were elevated on the 10^th^ DPT (p<0.05), then returned to their normal values on the 14^th^ DPT (p>0.05). In gentamicin 0.1% treated group, no significant change for RBCs values at different time points of treatment when compared with healthy ones (p>0.05), but Hb and PCV values were decreased on the 3^rd^ DPT than healthy ones (p<0.05), then returned to their normal values when compared to control ones (p>0.05; Table-3). Moreover, white blood cells (WBCs) and neutrophils were significantly increased in infected dogs on the 3^rd^-14^th^ day without treatment, while in dogs treated with *A. vera* gel ointment 20%, WBCs and neutrophils were elevated on the 3^rd^, 7^th^, and 10^th^ DPT (p<0.05) and returned to their normal values on the 14^th^ day when compared to healthy ones (Table-3). In dogs treated with *A. vera* gel ointment 20% and gentamicin ointment 0.1%, WBCs were increased on the 3^rd^, 7^th^, and 10^th^ DPT (p<0.05) and returned to their normal values on the 14^th^ day, except for neutrophils which were elevated on the 3^rd^ and 7^th^ day (p<0.05) then returned to their normal values on the 10^th^ and 14^th^ DPT when compared to healthy ones. Lymphocytes, monocytes, and eosinophils were decreased in all treated groups (p<0.05) and returned to their baseline on the 14^th^ DPT when compared to healthy groups (p>0.05; Table-3). Furthermore, mean corpuscular volume, mean corpuscular hemoglobin (MCH), and MCH concentration values were not significantly different between all groups except on the 3^rd^ DPT at which these values were decreased when compared with control ones (p<0.05; Table-3).

**Table-3:** Hematological profile of control and infected dogs. Values expressed as mean with SE.

Variables	RBCs (10^6^/µL)	Hb (g/dl)	PCV (%)	MCV (fi)	MCH (pg)	MCHC (%)	WBCs (10^3^/µL)	Neutrophil (%)	Lymphocyte (%)	Monocyte (%)	Eosinophil (%)
Control negative dogs (before induction) (n=20)	5.96±0.15^a^	13.5±0.3^a^	33.8±0.66^a^	56.75±0.7^a^	22.7±1.7^a^	40.1±1.7^a^	18.89±0.5^a^	61.75±0.62^a^	30.75±0.85^a^	5.5±0.28^a^	2±0.4^a^
Infected dogs without treatment Group 1 (n=5)											
3 DWT	5.5±0.11^b^	12.3±0.1^b^	31.5±0.6^b^	56.8±2.2^a^	22.1±0.2^a^	39.1±1.2^a^	30.47±0.6^b^	68.25±0.8^b^	26±0.57^b^	4.25±0.2^b^	1.5±0.28^b^
7 DWT	5.6±0.08^b^	12.6±0.08^b^	32±0.4^c^	57.1±0.12^a^	22.5±0.3^a^	39.4±0.5^a^	28.4±0.3^b^	65.7±0.4^c^	28±0.4^b^	4.5±0.28^b^	1.75±0.25^a^
10 DWT	5.6±0.06^b^	12.5±0.1^b^	32.7±0.4^c^	58.2±0.6^a^	22.2±0.2^a^	38.2±0.3^a^	26.39±0.4^c^	65±0.4^c^	28.5±0.6^b^	5±0.0^a^	1.5±0.28^b^
14 DWT	5.7±0.06^b^	12.7±0.09^b^	33±0.4^a^	56.9±1.5^a^	21.6±0.4^a^	38.4±0.7^a^	22.9±0.59^d^	63.5±0.6^d^	30.5±0.6^a^	4.75±0.25^b^	1.25±0.25^c^
Dogs treated with *Aloe vera* gel ointment 20% Group 2 (n=5)											
3 DPT	5.5±0.06^b^	11.3±0.2^c^	33±0.4^a^	59.1±0.17^b^	21.7±0.5^a^	36.7±0.7^b^	28.32±0.4^b^	66.25±0.47^c^	28.25±0.25^b^	4.2±0.25^b^	1.2±0.25^c^
7 DPT	6±0.09^a^	13.1±0.16^a^	33±1.08^a^	55.1±2.52^c^	21.2±0.58^a^	40±0.9^a^	25.15±0.38^c^	64.25±0.25^c^	29.75±0.25^a^	4.5±0.28^b^	1.5±0.28^b^
10 DPT	6.2±0.1^a^	13.47±0.2^a^	33.5±0.6^a^	54.1±1.82^c^	21.75±0.5^a^	40.23±0.5^a^	23.4±0.35^d^	62.75±0.47^a^	30.75±0.47^a^	5±0.4^a^	1.5±0.28^b^
14 DPT	6.06±0.05^a^	13.07±0.1^a^	33.75±0.6^a^	54.48±1.4^c^	21.1±0.44^a^	38.7±0.45^a^	20.34±0.57^a^	62.75±0.47^a^	30.75±0.47^a^	5±0.4^a^	1.5±0.28^b^
Dogs treated with *Aloe vera* gel ointment 40% Group 3 (n=5)											
3 DPT	6±0.09^a^	12.17±0.2^b^	32.5±0.9^a^	54.1±1.12^c^	20.26±1.2^b^	37.4±0.4^b^	28.2±0.35^b^	66.75±0.47^c^	27.25±0.25^b^	4.7±0.25^a^	1.2±0.25^c^
7 DPT	6.1±0.09^a^	13±0.09^a^	33.2±0.62^a^	54.5±0.98^c^	21.3±0.39^a^	39.1±0.86^a^	26.75±0.21^c^	65±0.47^c^	29±0.4^a^	4.5±0.288^b^	1.5±0.28^b^
10 DPT	6.15±0.13^a^	13±0.18^a^	35.2±0.47^d^	57.39±1.56^a^	21.17±0.6^a^	36.88±0.3^b^	24.7±0.44^d^	62±0.4^a^	31.25±0.47^a^	5±0.4^a^	1.75±0.25^a^
14 DPT	6.25±0.14^a^	13.05±0.1^a^	33.7±0.85^a^	54.4±2.12^c^	20.8±0.35^a^	38.76±1.3^a^	20.95±0.28^a^	61.5±0.28^a^	31.75±0.25^a^	5.25±0.25^a^	1.5±0.28^b^
Dogs treated by gentamicin sulfate 0.1% ointment Group 4 (n=5)											
3 DPT	5.7±0.19^a^	12.17±0.24^b^	30.5±0.64^b^	53.65±2.7^c^	21.36±0.9^a^	39.8±0.6^a^	30.1±0.38^b^	64±0.4^d^	30±0.4^a^	4.5±0.28^b^	1.5±0.28^b^
7 DPT	6.1±0.1^a^	13.3±0.29^a^	32.7±0.47^a^	53.7±1.38^c^	21.9±0.78^a^	40.8±1.2^a^	27.6±0.21^b^	63±0.4^d^	29.75±0.47^a^	5.25±0.25^a^	2±0.25^a^
10 DPT	6.12±0.08^a^	12.8±0.17^a^	32.25±0.8^a^	52.6±0.82^c^	21.01±0.2^a^	39.9±0.5^a^	23.125±0.5^d^	61.75±0.6^a^	31.25±0.47^a^	5.25±0.25^a^	1.75±0.25^a^
14 DPT	6.35±0.1^a^	13.1±0.19^a^	31.5±0.64^b^	49.6±1.7^d^	20.7±0.18^b^	41.9±1.3^a^	20.9±0.51^a^	62.5±0.2^a^	30.75±0.47^a^	5±0.4^a^	1.75±0.25^a^

SE=Standard error, DWT=Day without treatment, DPT=Day post-treatment, n=Number. Means with different letter superscripts in the same column are significantly different at p<0.05

### Oxidant and antioxidant concentrations between dogs’ studied groups

**Figure-4:**
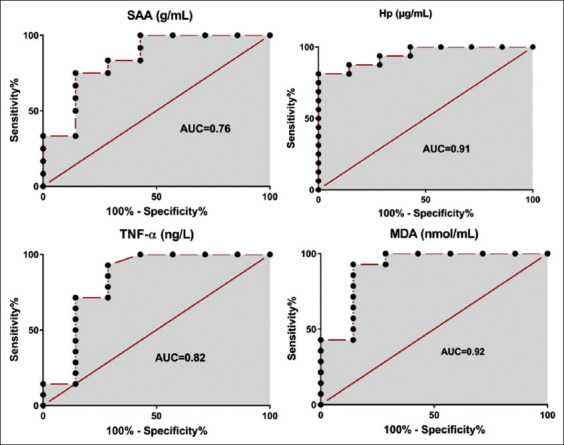
Receiver operating characteristic curve analysis for SAA, HP, TNF-α, and MDA concentrations in dogs with Staphylococcus pyoderma (p<0.05). SAA=Serum amyloid A, HP=Haptoglobin, TNF-α=Tumor necrosis factor-α, MDA=Malondialdehyde.

Serum MDA concentrations were higher in infected dogs on the 3^rd^-14^th^ day without treatment compared to control ones (p<0.05; Table-4), while dog treated with *A. vera* gel ointment 20% and 40% and gentamicin 0.1%, serum MDA concentrations were elevated on the 3^rd^ and 7^th^ DPT (p<0.05) and returned to its baseline on the 10^th^ and 14^th^ DPT compared to control ones (p>0.05; Table-4). Serum MDA concentrations were a diagnostic biomarker for Staphylococcus pyoderma in dogs (area under the curve=0.92; p<0.05; Figure-4). Serum TAC and catalase concentrations were decreased in infected dogs on the 3^rd^-14^th^ day without treatment compared to control group (p<0.05). In dogs treated with *A. vera* gel ointment 20%, serum TAC and catalase concentrations were decreased on the 3^rd^ and 7^th^ DPT (p<0.05) and returned to their normal concentrations on the 10^th^ and 14^th^ DPT compared to healthy ones (p>0.05). In dogs treated with *A. vera* gel ointment 40%, serum TAC and catalase concentrations were decreased on the 3^rd^ DPT (p<0.05) and returned to their baseline from the 7^th^ to14^th^ DPT when compared to healthy ones (p>0.05). In dogs treated with gentamicin 0.1%, serum TAC concentrations were decreased on the 3^rd^ and 7^th^ DPT (p<0.05) and returned to their baseline on the 10^th^ and 14^th^ DPT when compared to healthy ones (p>0.05), while catalase concentrations were not changed all over the time points than healthy ones (p>0.05; Table-4).

**Table-4:** Oxidant and antioxidant status of control and infected dogs. Values expressed as mean with SE.

Variables	MDA (nmol/mL)	TAC (mM/L)	Catalase (U/L)
Control negative (before induction) (n=20)	10.35±0.46^a^	1±0.06^a^	72.26±0.95^a^
Reference range of negative control	(3.20-17.80)	(0.40-1.30)	(52.3-100.40)
Infected dogs without treatment Group 1 (n=5)			
3 DWT	21.8±0.45^b^	0.41±0.1^b^	55.4±0.8^b^
7 DWT	22.2±0.14^b^	0.35±0.09^c^	53.7±1.2^c^
10 DWT	22.4±0.8^b^	0.32±0.04^c^	51.2±1.4^c^
14 DWT	23.8±0.23^c^	0.30±0.08^c^	50.8±0.54^c^
Dogs treated with *Aloe vera* 20% Group 2 (n=5)			
3 DPT	20.12±0.37^b^	0.597±0.03^d^	65.47±1.27^d^
7 DPT	19.78±0.54^b^	0.6±0.03^d^	67.3±0.5^d^
10 DPT	11.8±0.56^a^	0.83 ±0.07^a^	69.44±0.37^a^
14 DPT	12.45±0.29^a^	0.877±0.033^a^	69.71±0.3^a^
Dogs treated with *Aloe vera* 40% Group 3 (n=5)			
3 DPT	19.56±0.42^b^	0.707±0.033^d^	66.97±0.63^d^
7 DPT	16.4±0.4^d^	0.905±0.055^a^	68.99±0.56^a^
10 DPT	11.73±0.37^a^	0.916±0.032^a^	70.36±0.4^a^
14 DPT	11.8±0.22^a^	0.882±0.047^a^	69.45±0.44^a^
Dogs treated by gentamicin sulfate 0.1% Group 4 (n=5)			
3 DPT	16.24±0.29^d^	0.67±0.025^d^	70.125±0.64^a^
7 DPT	15.13±0.42^d^	0.64±0.04^d^	71.03±0.56^a^
10 DPT	12.43±0.26^a^	0.88±0.04^a^	70.10±0.2^a^
14 DPT	12.12±0.19^a^	0.98±0.04^a^	71.5±0.58^a^

SE=Standard error, MDA=Malondialdehyde, TAC=Total antioxidant capacity, DWT=Day without treatment, DPT=Day post-treatment, n=Number. Means with different letter superscripts in the same column are significantly different at p<0.05

### Acute-phase proteins and cytokines concentrations between studied groups of dogs

There was a significant increase in the concentrations of acute-phase proteins (CP, SAA, and HP) and ILs (IL-1, IL-6, IL-10, and TNF-α) in infected dogs on the 3^rd^-14^th^ day without treatment and in dogs treated with *A. vera* gel 20% and 40% and gentamycin ointments 0.1% on the 3^rd^ and 7^th^ DPT when compared to the control negative ones (p<0.05; Table-5), then started to return to their baseline on the 14^th^ DPT in dogs treated with *A. vera* gel ointment 20% and 40% and gentamicin 0.1% when compared to control ones (p>0.05). SAA, HP, and TNF-α concentrations were significant biomarkers for Staphylococcus pyoderma in dogs (p<0.05; Figure-4). Serum HP and TNF-α concentrations were significantly lower on the 10^th^ and 14^th^ DPT in dogs treated with *A. vera gel* 20% and 40% compared to dogs treated with gentamycin 0.1% (p<0.05; Table-5). Dogs’ pyoderma treated with *A. vera* 20% and 40% were more likely to have low HP and TNF-α concentrations than gentamicin ([OR=4.6; 95% CI=1.31-17.40; p<0.05]; [OR=5.2; 95% CI=1.04-22.30; p<0.05]), respectively, as shown in Table-6.

**Table-5:** Acute-phase protein biomarkers and cytokines expression in control and infected dogs. Values expressed as mean with SE.

Variables	Ceruloplasmin (µg/mL)	Serum amyloid A (g/mL)	Haptoglobin (µg/mL)	Interleukin 1 (pg/mL)	Interleukin 6 (ng/L)	Interleukin 10 (ng/L)	Tumor necrosis factor-α (ng/L)
Control negative (before induction) (n=5)	25.8±0.24^a^	0.62±0.04^a^	67.25±2.2^a^	59.3±0.6^a^	29.5±0.8^a^	40.5±0.4^a^	12.04±0.2^a^
Reference range of negative control	(10.2-40.2)	(0.2-0.76)	(58.30-85.20)	(42.3-70.50)	(21.3-40.2)	(35.2-48.4)	(10.25-15.35)
Infected dogs without treatment Group 1 (n=5)							
3 DWT	57.38±0.2^b^	1.8±0.8^b^	132.7±1.8^b^	101.4±1.6^b^	42.4±0.6^b^	58.5±0.8^b^	25.3±0.2^b^
7 DWT	51.51±0.3^c^	1.6±0.1^b^	128.4±2.4^b^	100.8±0.78^b^	40.7±1.2^b^	57.4±1^b^	20.8±0.4^c^
10 DWT	50.8±0.08^c^	1.5±0.06^b^	125.6±1.6^b^	90.8±0.5^c^	38.8±0.6^b^	48.8±0.8^c^	18.8±0.2^c^
14 DWT	44.54±0.5^d^	0.8±0.03^c^	124.7±1.2^b^	85.2±2.2^c^	35.2±0.8^c^	45.6±0.6^c^	18.2±0.5^c^
Dogs treated with *Aloe vera* gel ointment 20% Group 2 (n=5)							
3 DPT	48.45±0.65^c^	1.4±0.2^b^	118.8±2.2^b^	88.8±0.8^c^	38.2±1.4^b^	54.3±1.4^b^	18.2±0.4^c^
7 DPT	42.8±0.4^d^	0.9±0.08^c^	82.54±1.2^c^	74.2±1.4^d^	35.8±0.5^c^	42.4±0.6^a^	15.8±0.6^d^
10 DPT	29.3±0.8^a^	0.85±0.05^c^	72.2±1.8^c^	64.5±0.6^a^	32±0.8^a^	42.5±0.8^a^	13.2±0.8^a^
14 DPT	28.12±0.54^a^	0.68±0.1^a^	70.6±1.1^a^	60.3±1.2^a^	30.8±0.6^a^	41.8±0.5^a^	12.5±0.2^a^
Dogs treated with *Aloe vera* gel ointment 40% Group 3 (n=5)							
3 DPT	52.4±0.3^c^	1.4±0.1^b^	115.4±2.4^b^	85.2±0.95^c^	38±0.38^b^	55.3±0.7^b^	19.2±0.5^c^
7 DPT	44.48±0.38^d^	1.08±0.06^c^	80.7±1.2^c^	73.6±0.8^d^	32.4±0.8^a^	42.8±0.3^a^	15.7±0.2^d^
10 DPT	30.2±0.4^a^	0.8±0.0^c^	73.8±0.8^a^	65.2±0.4^a^	30.4±0.4^a^	42±0.6^a^	13.6±0.8^a^
14 DPT	28.7±0.6^a^	0.66±0.04^a^	70.85±1.2^a^	61.4±1.2^a^	30±1.3^a^	40.6±0.9^a^	12.2±0.6^a^
Dogs treated by gentamicin sulfate 0.1% sulfate 0.1% ointment Group 4 (n=5)							
3 DPT	48.12±0.2^c^	1.28±0.1^c^	116.6±1.4^b^	87.9±0.4^c^	39.2±0.6^b^	55.8±0.6^b^	18.4±0.2^c^
7 DPT	44.2±0.4^d^	1.18±0.06^c^	112.9±0.8^b^	74.8±1.2^d^	38.5±0.8^b^	45.8±0.8^c^	16.2±0.3^d^
10 DPT	42.42±0.5^d^	0.85±0.02^c^	111.4±1.2^c^	68.4±0.58^a^	32.2±0.5^a^	40.6±1.08^a^	19.6±0.4^d^
14 DPT	30.4±0.6^a^	0.70±0.04^a^	112.4±2.2^c^	63.42±0.6^a^	31.4±1.4^a^	40.2±0.7^a^	19.8±0.2^d^

SE=Standard error, n=Number. Means with different letter superscripts in the same column are significantly different at p<0.05

**Table-6:** Univariate logistic regression of variables in dogs’ staphylococcal pyoderma.

Variables	Tested drugs	Range	OR	95% CI	p-value
HP (µg/mL)	*Aloe vera* gel ointment 20% and 40% Gentamicin ointment 0.1%	58.3-85.2 >85.2	4.6*	1.31-17.40	0.03
TNF-α (ng/L)	*Aloe vera* gel ointment 20% and 40% Gentamicin ointment 0.1%	10.25-15.35 >15.35	Referent 5.2*	1.04-22.30	0.02

HP=Haptoglobin, TNF-α=Tumor necrosis factor-α, OR=Odds ratio, CI=Confidence interval.

* p<0.05 compared to referent

## Discussion

In the present study, the antibacterial effect of *A. vera* gel ointments 20% and 40% against dogs’ staphylococcal pyoderma had been evaluated *in vitro*. *A. vera* extract showed clear inhibition zone for *S. aureus* growth in well diffusion agar test. The inhibition zone of *A. vera* extract 40% was wider than the ones that caused by *A. vera* extract 20% and gentamicin 0.1%. Our results were in the same line with previous studies that recorded the antibacterial effect of *A. vera* extract against *S. aureus* [21,22]. The antibacterial activity of *A. vera* could be attributed to its components that include p-coumaric, ascorbic, pyrocatechol, and cinnamic acid that could be used as an alternative herbal antibacterial therapy to subside the antibiotic resistance and reduce wound healing time [22,23].

In the present study, RBCs, Hb, PCV and WBCs were returned to their normal values after topical application of different concentrations of *A. vera* gel ointments 20% and 40% on dogs’ pyoderma thus could be explained by *A. vera* may increase erythropoiesis process and also increase leukocyte infiltration as a part of immunomodulation [24].

We have shown that the concentrations of acute-phase proteins are in positive correlation with the severity of dog’s pyoderma that could be attributed to increase in their synthesis and release from the liver in response to tissue injury of skin [25]. Furthermore, we have documented that ILs were elevated in infected dogs than control ones. It is possible that pyoderma is suggested to increase an inflammatory response by releasing cytokines into the bloodstream [26,27]. Interestingly, the serum concentrations of MDA were significantly increased, while the TAC and catalase concentrations were decreased in infected dogs with staphylococcal pyoderma compared to control ones. It has been reported that dogs with skin pathological conditions have significant oxidative stress with elevated MDA and decreased TAC and catalase concentrations as reported in our study [28-30].

In the current study, topical application of *A. vera* gel ointment 20% and 40% modulates the inflammatory response through reduction of acute-phase proteins and IL concentrations and enhances skin healing of dogs’ Staphylococcus pyoderma. The previous studies documented that topical application of different doses of *A. vera* extract on injured skin resulting in reduction of the acute-phase response [31,32]. Moreover, the positive effect of *A. vera* gel on oxidant and antioxidant status has been shown in this study through elevation TAC and catalase concentrations and reduction of MDA concentrations that could be explained potentially by *A. vera* had antioxidant effects through glutathione peroxidase activity [33]. From that prospective, one could speculate that *A. vera* gel ointment has anti-inflammatory and antioxidative roles against dogs’ staphylococcal pyoderma.

## Conclusion

Increased acute-phase proteins, cytokines, and oxidants and reduced antioxidant biomarkers were frequent findings in dog’s pyoderma. Furthermore, topical application of *A. vera* gel ointments 20% and 40% had antibacterial, anti-inflammatory, and antioxidative actions against dogs’ staphylococcal pyoderma that will support its further use rather than antibiotics in the health arena.

## Authors’ Contributions

HH designed the experiment. AA, AhE, AE, and HK performed the experiment. AK performed the statistical analysis of results. HH, AK, and AA drafted and revised the manuscript. All authors read and approved the final manuscript.
